# GPR68 Contributes to Persistent Acidosis-Induced Activation of AGC Kinases and Tyrosine Phosphorylation in Organotypic Hippocampal Slices

**DOI:** 10.3389/fnins.2021.692217

**Published:** 2021-05-25

**Authors:** Guokun Zhou, Xiang-ming Zha

**Affiliations:** Department of Physiology and Cell Biology, University of South Alabama College of Medicine, Mobile, AL, United States

**Keywords:** neuronal injury, acid signaling, GPR68 (OGR1), phosphorylation, hippocampal slice

## Abstract

Persistent acidosis occurs in ischemia and multiple neurological diseases. In previous studies, acidic stimulation leads to rapid increase in intracellular calcium in neurons. However, it remains largely unclear how a prolonged acidosis alters neuronal signaling. In our previous study, we found that GPR68-mediated PKC activities are protective against acidosis-induced injury in cortical slices. Here, we first asked whether the same principle holds true in organotypic hippocampal slices. Our data showed that 1-h pH 6 induced PKC phosphorylation in a GPR68-dependent manner. Go6983, a PKC inhibitor worsened acidosis-induced neuronal injury in wild type (WT) but had no effect in GPR68^−/−^ slices. Next, to gain greater insights into acid signaling in brain tissue, we treated organotypic hippocampal slices with pH 6 for 1-h and performed a kinome profiling analysis by Western blot. Acidosis had little effect on cyclin-dependent kinase (CDK) or casein kinase 2 activity, two members of the CMGC family, or Ataxia telangiectasia mutated (ATM)/ATM and RAD3-related (ATR) activity, but reduced the phosphorylation of MAPK/CDK substrates. In contrast, acidosis induced the activation of CaMKIIα, PKA, and Akt. Besides these serine/threonine kinases, acidosis also induced tyrosine phosphorylation. Since GPR68 is widely expressed in brain neurons, we asked whether GPR68 contributes to acidosis-induced signaling. Deleting GPR68 had no effect on acidosis-induced CaMKII phosphorylation, attenuated that of phospho-Akt and phospho-PKA substrates, while abolishing acidosis-induced tyrosine phosphorylation. These data demonstrate that prolonged acidosis activates a network of signaling cascades, mediated by AGC kinases, CaMKII, and tyrosine kinases. GPR68 is the primary mediator for acidosis-induced activation of PKC and tyrosine phosphorylation, while both GPR68-dependent and -independent mechanisms contribute to the activation of PKA and Akt.

## Introduction

Protons regulate multiple biological processes, including energy metabolism, synaptic plasticity, and neuronal survival in the brain (Chesler, 2003; Huang Y. et al., 2015). A persistent increase in extracellular proton concentration, or acidosis, in the brain occurs in diseases including ischemic stroke, recurrent seizures, and traumatic brain injury. The reduction in extracellular pH can range from a few tenths of units to below pH 6.0 (Huang Y. et al., 2015). Through direct activation of proton-sensitive receptors and indirect modulation of other proteins, acidosis can activate multiple signaling pathways in various cell types (Yamaji et al., 1997; Beauloye et al., 2001; Rohra et al., 2004; Xiong et al., 2004; Yermolaieva et al., 2004; Chen et al., 2011; Xu et al., 2018; Krewson et al., 2020). However, most of the neuronal study focuses on acid-induced acute rise of calcium (Xiong et al., 2004; Yermolaieva et al., 2004; Zha et al., 2006; Wang et al., 2020b). The majority of acidosis-induced, calcium-independent, signaling was discovered in non-neuronal cells. Revealing how persistent pH reduction alters signaling cascades in neuronal tissue will provide crucial insights into our understanding of acid signaling in multiple diseases.

Brain expresses several types of proton receptors, including the acid-sensing ion channel−1a (ASIC1a) and−2 (ASIC2), proton-activate chloride channel (PAC; TMEM206), GPR4, GPR65, and GPR68 (Wemmie et al., 2002; Askwith et al., 2004; Xiong et al., 2004; Zha et al., 2009; Sherwood et al., 2011; Du et al., 2014b; Price et al., 2014; Wei et al., 2015; Guyenet et al., 2016; Hosford et al., 2018; Ullrich et al., 2019; Yang et al., 2019; Sato et al., 2020; Wenzel et al., 2020; Zhou et al., 2021). ASIC1a, PAC, and GPR68 exhibit neuronal expression in the brain. GPR4, which can activate multiple downstream signaling pathways, is high in blood vessel and has relatively limited expression in neurons. GPR65, which couples to Gs, exhibits limited expression in some microglia in the brain. Among the three receptors which exhibit predominant neuronal expression, ASIC1a leads to calcium influx while PAC conducts chloride current; both leads to neuronal injury (Xiong et al., 2004; Yermolaieva et al., 2004; Yang et al., 2019). In contrast, GPR68 activation leads to neuroprotection (Wang et al., 2020a,b). Our recent study showed that GPR68 mediates acidosis-induced PKC activation (Wang et al., 2020b). Together, these data demonstrate that acidosis can activate multiple receptors in the brain. To gain greater insights into acidosis in brain physiology and disease, it will be necessary to better define acid-induced signaling in neuronal tissue. In addition, to better interpret the protective role of GPR68, it will be necessary to determine the contribution of GPR68.

In this study, we examined organotypic hippocampal slices, which maintain the 3D architecture and complexity of the *in vivo* tissue. To determine whether GPR68-PKC axis regulates acidotoxicity in hippocampal slices, we first assessed the effect of PKC signaling and its inhibition on acidosis-induced neuronal injury. Next, we analyzed changes in kinome activities using phosphorylation-specific antibodies and Western blot. Lastly, we focused on acidosis-activated pathways and asked whether GPR68 mediates the activation.

## Materials and Methods

### Mice

The GPR68^−/−^ mice (MGI Cat# 3849480, RRID:MGI:3849480) have been previously described and were maintained on a congenic C57BL/6 background (Li et al., 2009). Wild-type C57BL/6 and GPR68^−/−^ mice were maintained as breeding colonies at the University of South Alabama. According to the Jackson Laboratory's recommendations, the GPR68^−/−^ mice were refreshed (backcrossed to C57BL/6 wild-type) every 5–10 generations. Animals were housed 2–4 in a cage with *ad libitum* access to chow and water, under temperature-controlled conditions on a 12 h light/dark cycle. Animal care met National Institutes of Health standards. For slice cultures, post-natal day 5–7 (P5–7) pups (either sex) were used. The protocol for animal usage was reviewed and approved by the Animal Care and Use Committee at University of South Alabama.

### Antibodies and Reagents

Table 1 lists the primary antibodies used. The mouse monoclonal anti-CaMKIIα was a gift from Dr. Johannes Hell. Secondary antibodies used: IRDye-680/700- or 800-, or DyLight-680/700- and 800- conjugated secondary antibodies (ThermoFisher Invitrogen, Li-Cor, or Rockland Inc.). Cell culture media and serum were purchased from TheromoFisher.

**Table 1:** List of commercial primary antibodies used for Western blot.

**Target (host)**	**Phosphorylation site**	**Dilution**	**Company**	**Catalog #**
Phospho-Akt Substrate (rabbit)	RXX(S*/T*)	1:2,000	Cell Signaling Technology	9614S
Phospho-(Ser/Thr)ATM/ATRSubstrate (rabbit)	(S*/T*)QG, (S*/T*)Q	1:2,000	Cell Signaling Technology	6966S
Phospho-(Ser) CDK Substrate (rabbit)	(K/H)S*P	1:2,000	Cell Signaling Technology	8739S
Phospho-(Ser/Thr) CK2 Substrate (rabbit)	(S*/T*)DXE	1:2,000	Cell Signaling Technology	8738S
Phospho-MAPK/CDK Substrates (rabbit)	PXS*P, S*PX(K/R)	1:2,000	Cell Signaling Technology	2325S
Phospho-PKA Substrate (rabbit)	(K/R)(K/R)X(S*/T*)	1:2,000	Cell Signaling Technology	9624S
Phospho-(Ser) PKC Substrate (rabbit)	(K/R)XS*X(K/R)	1:2,000	Cell Signaling Technology	6967S
Phospho-Thr-X-Arg Motif (rabbit)	T*X(K/R)	1:2,000	Cell Signaling Technology	2351S
Phospho-CaMKIIα (rabbit)	Thr286	1:2,000	Cell Signaling Technology	12716S
Phospho-tyrosine (rabbit)	pTyr	1:2,000	Cell Signaling Technology	6967S
β-tubulin (mouse)		1:60k	Sigma	T4026

### Organotypic Hippocampal Slice Culture and Treatment

Post-natal day 5–7 pups (either sex) were rapidly decapitated. Organotypic hippocampal slices from WT and GPR68^−/−^ mice were cultured as described previously (Zha et al., 2006; Jing et al., 2012). Briefly, slices (350 μm thick) were cut with a tissue chopper and the intact slices were randomly grouped before plating. The slices were then transferred onto Falcon polyethylene terephthalate-etched membrane culture inserts with 1 μm pores (Fisher) in 6 well plates, 5–6 slices per insert. Each well contains 1.2 ml FCM (25% HBSS, 25% horse serum and 50% MEM with 2 mM Glutamax, 1.5 mg/ml glucose, 5 U/ml pen/strep and supplemented with extra 4.5 mM NaHCO_3_). Slice cultures were kept in a humidified CO_2_ incubator maintained at 37°C 5.5% CO_2_ for 9–12 days. Medium was changed every 2–3 days.

In our study, the genotype was not blinded to the experimenter. However, we randomly assigned the filters from the same animal (culture) to different treatment conditions. Thus, the pH 7.4 condition in each culture (from the same animal) serves as the internal control for phosphorylation or survival baseline (i.e., at pH 7.4). This practice controls for the variation in slice preparation from animal to animal. At 9 or 12 days *in vitro* (in culture), the cultured WT or GPR68^−/−^ hippocampal slices transwells were subject to different pH treatment groups. The pH medium was prepared as: HBSS supplemented with 1% horse serum, 1 × essential amino acid (Invitrogen), 1 × vitamin mix (Invitrogen), 1 × Glutamax, and 1.5 mg/ml glucose. To buffer pH, we used 20 mM HEPES for pH 7.4 and 20 mM MES for pH 6.0. On the treatment day, pH medium was warmed to 37°C. To ensure that the medium pH is stable during the treatment, we measured the medium pH with a pH meter, and adjusted pH with 1N HCl or NaOH at least 2 times with ~30 min interval before treatment. Slices were then incubated in CO_2_ incubator for 60 or 120 min as indicated.

### Acidosis-Induced Injury and Imaging

Slice injury analysis in organotypic hippocampal slices was performed similar to earlier studies (Jiang et al., 2017; Wang et al., 2020b). Briefly, at 22–24 h following the treatment, 1 μl of Syto-13 and 13.5 μl of propidium iodide (1 mg/ml) were diluted with 2.5 ml FCM. 0.4 ml of this medium was mixed with the culture medium (1.2 ml) in the culture plate, and 0.8 ml of the resultant staining medium was added on top of the culture insert. After 1 h incubation, 0.8 ml of the staining medium in the well was transferred again to the top of the insert, and incubated for another hour. Slices were rinsed once by adding 1 ml of ice-cold HBSS with calcium and magnesium (HBSS+/+) containing 6 mg/ml glucose to the top of the insert. The inserts were kept for 20–30 min on ice or at 4°C to allow the HBSS buffer to pass through the insert. The 20–30 min incubation time was to allow sufficient time for the solution to pass the thick slice. Doing so reduced the amount of dyes which did not enter the cells and thus reduced background fluorescence from dyes in extracellular space. Since the slices were kept on ice and we fixed the slices right after this rinse step, the incubation itself did not appear to induce increased injury, as evidenced by low propidium iodide signals in the pH 7.4 group. Following fixation for 15–30 min with 4% paraformaldehyde, the slices were imaged as described in previous studies (Jiang et al., 2017; Wang et al., 2020b). For the time-dependent experiment, we imaged one set of experiment with confocal microscopy and another set with conventional fluorescent microscope with SPOT Imaging. The two sets of images generate similar changes (i.e., no injury at 1-h pH 6) and the data were combined and presented in the first figure. For survival experiment using Go6983, all three sets of experiments were imaged with a conventional Olympus IX70 fluorescent microscope.

### Acute Hippocampal Slice Preparation and Treatment

Acute hippocampal slices were prepared from 7 to 9-week-old male mice, similar to earlier descriptions (Xu et al., 2020). Briefly, mice were anesthetized and rapidly decapitated. The brain was quickly removed and placed in ice-cold sucrose-containing artificial cerebrospinal fluid (ACSF) (in millimolar: 70 sucrose, 80 NaCl, 2.5 KCl, 21.4 NaHCO_3_, 1.25 NaH_2_PO_4_, 0.5 CaCl_2_, 7 MgCl_2_, 1.3 ascorbic acid, and 20 glucose). Transverse hippocampal slices (350 μm) were cut with slice chopper and transferred into a holding chamber containing ACSF (in millimolar: 125 NaCl, 2.5 KCl, 24 NaHCO_3_, 1.25 NaH_2_PO_4_, 2.0 CaCl_2_, 1.0 MgCl_2_, and 15 glucose). Slices were incubated at 35°C for 30 min and then at room temperature for 1 h before pH treatment. All solutions were constantly bubbled with 95% O_2_ and 5% CO_2_. For pH treatment, ACSF plus 20 mM HEPES (for pH 7.4) or MES (for pH 6.0) was used. The pH was adjusted by adding HCl or NaOH, and re-checked/re-adjusted several times until pH no longer drifts between adjustments. One technical note is that, probably due to the starting higher level of bicarbonate, the pH of ACSF-based solution typically took longer time to stabilizes as compared to that of HBSS (which has 4.2 mM of bicarbonate) used for organotypic slice treatment. For treatment, slices were transferred from the holding chamber to pH 6 for the indicated time, then transferred to an Eppendorf tube on ice until lysate preparation. Each animal generated enough slices for one time course experiment.

### Lysate Preparation and Western Blot Analysis

At the end of treatment period, organotypic slices were scraped off from the culture inserts using a 1 ml pipette and 200–300 μl of lysis buffer (PBS with 1% Triton X-100, 0.5% SDS, with freshly added protease inhibitors and phosphatase inhibitors). For acute slices, 2 slices were combined for one treatment condition and lysed with lysis buffer. Lysates were sonicated briefly and cleared by centrifugation for western blot analysis. Before loading, 1/2 volume of 3x SDS sample buffer was added to the lysates, and incubated at ~50°C for 15–20 min. The samples were separated by 10% SDS-PAGE and transferred to nitrocellulose membranes. Blotting was performed according to instructions of the Odyssey Imaging System (Li-Cor), similar to our previous studies (Jing et al., 2012; Xu et al., 2018). Briefly, membranes were blocked in blocking buffer (0.1% casein in 0.2 × PBS, pH 7.4) for 1 h. Primary antibodies were diluted with blocking buffer containing 0.1% Tween-20 and incubated at 4°C overnight. Secondary antibodies were diluted in blocking buffer containing 0.1% Tween-20 and 0.01% SDS and incubated at room temperature for 1 h. The primary antibodies dilutions were listed in Table 1. Secondary antibodies were IRDye 800CW Donkey anti-Rabbit 1:10,000 and IRDye 680LT Donkey anti-Mouse 1:10,000. Blots were imaged using an Odyssey Infrared Imaging System according to manufacturer's instructions. Densitometry of imaged bands was performed in NIH ImageJ as described earlier (Jiang et al., 2017).

### Statistical Analysis

No statistical method was used to determine sample size. Sample size was arbitrarily set to > =8 (2–4 cultures with 4–5 filters each) for survival studies and > =4 for phosphorylation studies. For phosphorylation in organotypic slices, we cultured each pup separately. One pup typically generated enough slices for 2–3 filter inserts, which were used for one experiment with no replicates within the experiment/culture. On the treatment day, we randomly assigned inserts from the same culture to treatment conditions. pH 7.4 in the same culture (i.e., the inserts obtained from the same animal) was used as the normalization control for the corresponding pH 6-treated filter. Thus, each dot in the Western blot figures corresponds to one data point generated from one individual animal. For phosphorylation in acute hippocampal slices, each animal generated enough slices for one time course. We used a total of 4 animals for the acute slice study. For survival analysis in organotypic slices, we used multiple pups per culture and the slices were pooled together, randomly mixed, and plated into filter inserts. Thus, each culture in survival assay used multiple animals. For survival, we imaged each slice separately. Each dot on the survival plot in figures represents the quantification from one slice. For analysis, statistics was performed in GraphPad Prism. There was no exclusion of outliers in our study. All data are presented as the mean ± S.E.M. Comparison between 2 groups was assessed by 2-tailed Student's *t*-test. Multiple comparisons were performed using one-way ANOVA with *post-hoc* Tukey's test. For time course experiment, all time points were compared with the pH 7.4 (time 0) control, using one-way ANOVA with Dunnett's *post-hoc* correction. Values were considered significant at *p* < 0.05.

## Results

### pH 6-Induced Neuronal Injury in Organotypic Hippocampal Slices Is Time-Dependent

**Figure 1:**
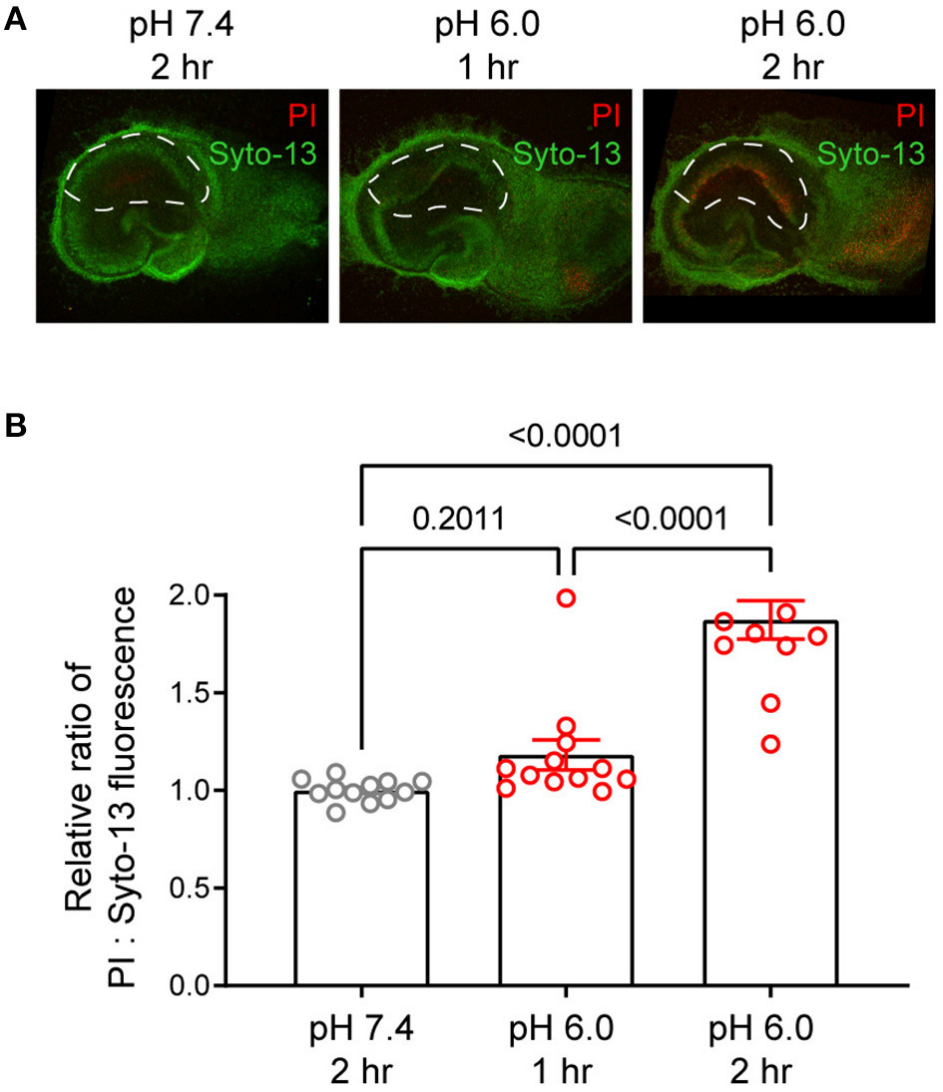
Time-dependent effect of pH 6 on neuronal injury. Organotypic hippocampal slices were treated with medium buffered at pH 7.4 or pH 6.0 for 1- or 2-h as indicated. To determine delayed neuronal injury, slices were stained 24 h later with propidium iodide (PI, red) and Syto-13 (green) as described in Methods, and imaged by fluorescence microscopy. **(A)** Representative confocal images and **(B)** Quantification of PI:Syto-13 fluorescence ratio. Increased red:green ratio indicates increased neuronal injury. Each dot represents one slice imaged from two separate experiments. ****p < 0.0001. *P*-values were from 2-way ANOVA with Tukey's correction.

Prolonged acidosis leads to neuronal injury. To focus on chronic acidosis-induced signaling without the confounding factor of neuronal injury, we first performed a time course analysis of neuronal injury in response to pH 6 in organotypic hippocampal slices. We treated the slices with medium buffered at either pH 7.4 or 6.0 for 1 or 2 h, stained with propidium iodide for neuronal injury 24 h later, and imaged with a fluorescent or confocal microscope (Jiang et al., 2017; Wang et al., 2020b). Consistent with previous reports, 2-h pH 6 induced injuries in CA1 pyramidal cell body layers (Figures 1A,B). In contrast, 1-h pH 6 had no apparent increase in propidium iodide staining, which indicates little induction of neuronal injury. For this reason, we used the 1-h protocol in this study to investigate persistent acidosis-induced signaling.

### PKC Inhibition Exacerbates Acidosis-Induced Neuronal Injury in Hippocampal Slices

**Figure 2:**
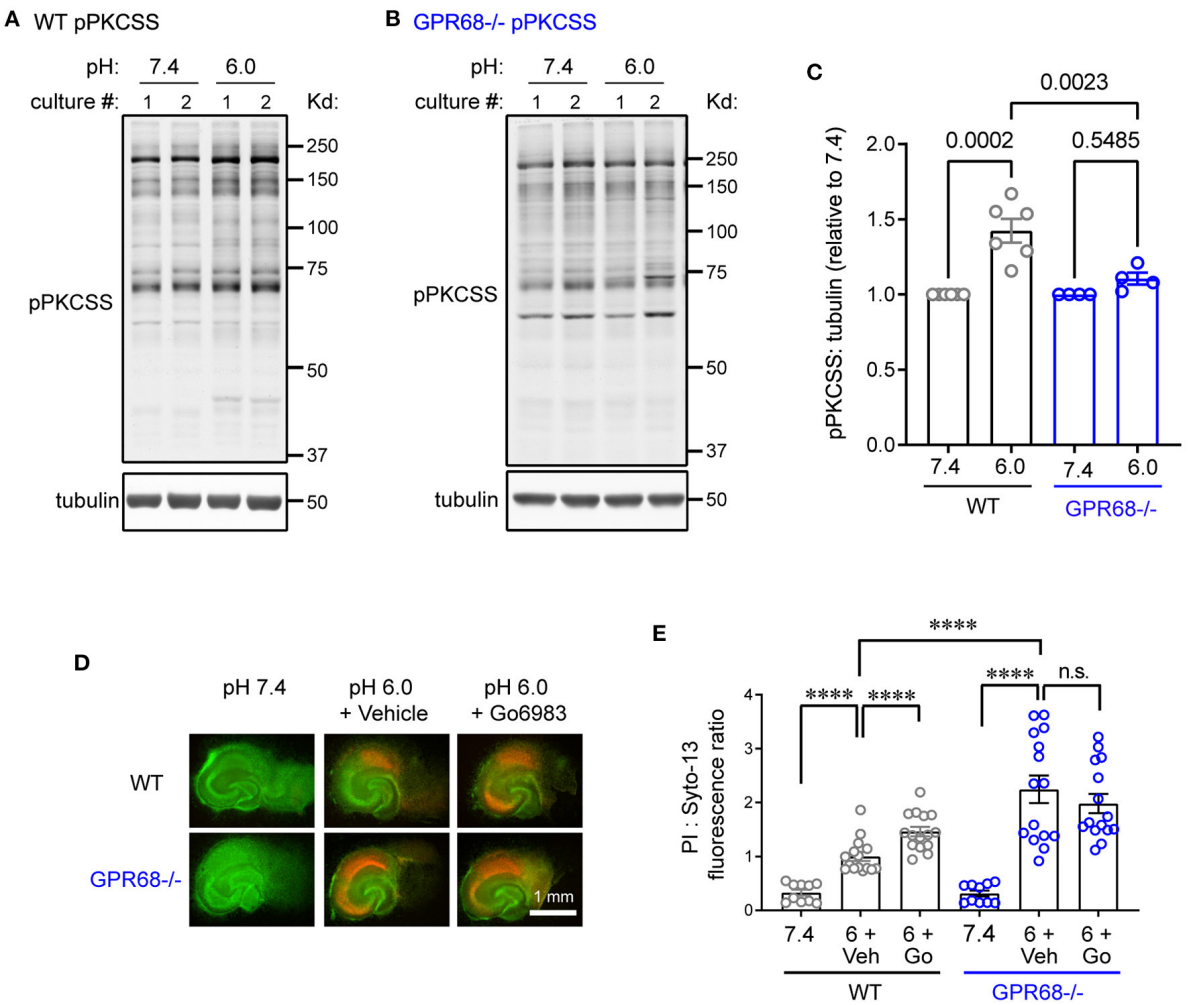
PKC inhibition on acidosis-induced neuronal injury. **(A–C)** Representative Western blot and quantification of 1-h pH 6-induced phosphorylation of PKC substrates (pPKCSS) in WT and GPR68^−/−^ hippocampal slices. As described in Methods, for phosphorylation, each animal (pup) generated one culture. The pH 6-treated group was normalized to the 7.4 control in the same culture (generated from the same animal). Each dot on the summary plot thus represents results obtained from one culture, which was generated from one animal. In Western blot analysis, for motif antibodies, we quantified the total number of pixels for the entire lane. **(D,E)** Representative fluorescence images **(D)** and quantification **(E)** of pH 6.0-induced neuronal injuries in organotypic hippocampal slices. Slices were treated for 2-h with pH 7.4 (control) or pH 6.0 in the presence of 0.05% DMSO (vehicle) or 5 μM Go6983. Neuronal injury was analyzed as described in Figure 1. As described in Methods, for survival analysis, each culture used slices isolated from multiple animals (pups). The slices were randomly plated in culture inserts and each slice was imaged separately. Each culture generates 5–6 slices per condition. Summary plot were pooled data from 3 separate experiments, with each dot represents one individual slice. *P*-values were from 2-way ANOVA with Tukey's correction. ****p < 0.0001.

In our previous study, we examined cortical slices and found that acidosis induced PKC activation through GPR68. To determine whether this is the case in hippocampal slices, we treated these slices with pH 6 for 1 h and blotted with an antibody recognizing phospho-PKC substrates (pPKCSS). pH 6 treatment led to significant increase in pPKCSS signal (Figure 2A). Similar to our previous result, GPR68^−/−^ slices did not exhibit significant increase in pPKCSS after pH 6 (Figures 2B,C). Next, to determine whether PKC activities mediate a protective function, we performed an *in vitro* neuronal injury analysis. We treated the organotypic hippocampal slices with pH 7.4 or 6.0 for 2 h, in the presence of either vehicle or Go6983, which inhibits PKC. Similar to our previous observation, Go6983 exacerbated pH 6-induced neuronal injury in WT slices (Figures 2D,E). In contrast, Go6983 had little effect in GPR68^−/−^ slices.

### Acidosis Has Little Effect on CMGC and ATM/ATR Kinases

**Figure 3:**
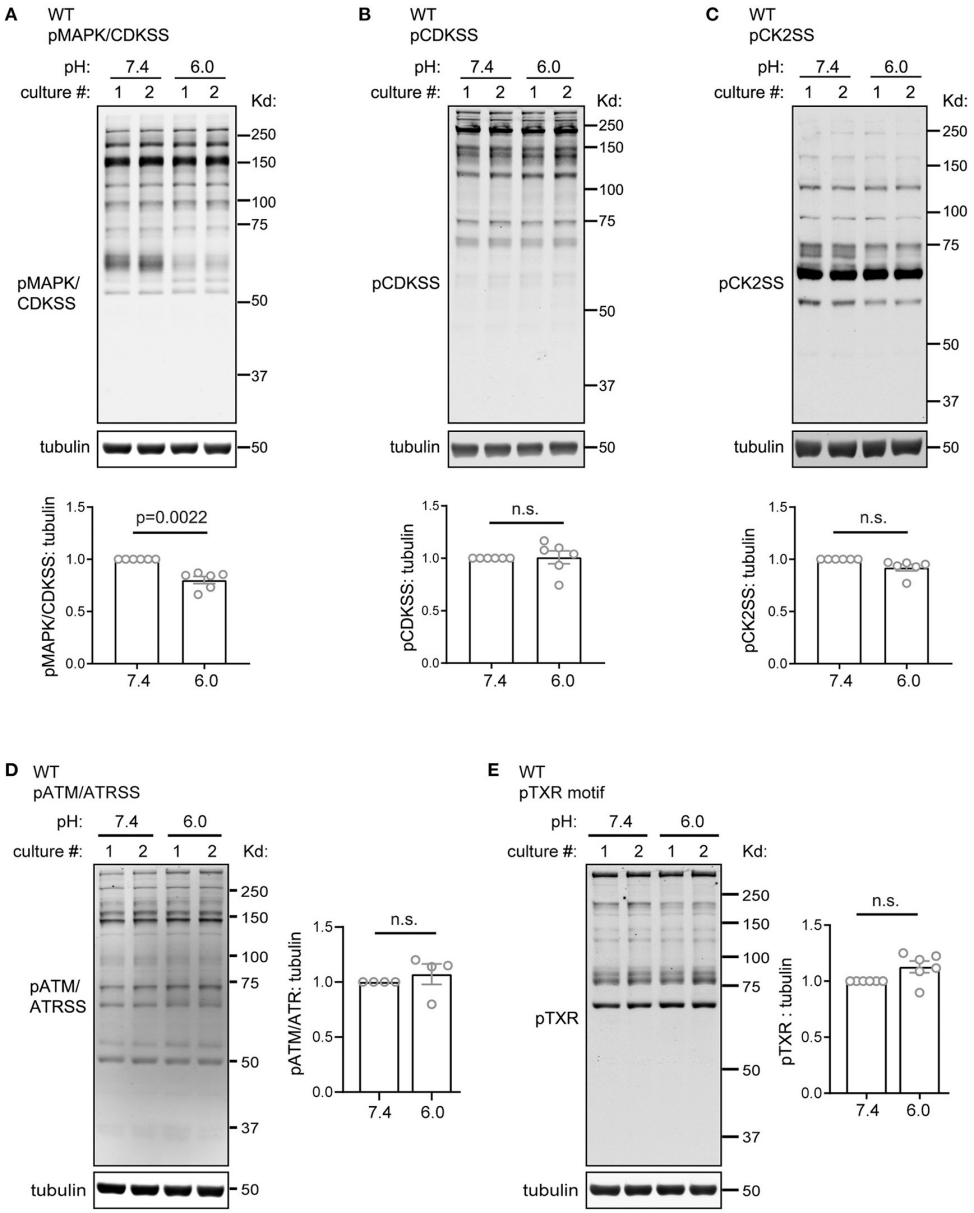
Acidosis had little effect on phosphorylation of substrates of CMGC kinases, ATM/ATR substrates, and phospho-TXR motif. Representative Western blot and quantification of pH 6.0-induced immunoreactivity of **(A)** pMAPK/CDK substrates (pMAPKSS), **(B)** pCDK substrates (pCDKSS), **(C)** pCK2 substrates (pCK2SS), **(D)** ATM/ATR substrates, and **(E)** phospho-TXR motif. Organotypic hippocampal slices were treated with pH media as indicated, lysed, and analyzed by Western blot. Tubulin was used as loading control. Blots shown were from two separate culture, as indicated on the top of the lanes. For quantification, pH 6 was normalized to the 7.4 condition from the same culture. Blots and images were representative from 4 to 6 different cultures/pups (see Methods or Figure 2 Legends for additional clarification). *P*-values were from 2-tailed Student's *t* test.

To gain better understanding of acid signaling in slices, we performed additional Western blot analysis using antibodies which detect the activation of various kinase pathways. We first examined the several members of the CMGC kinase family, which plays important roles in cell cycle, proliferation, differentiation, glycogen metabolism, transcription control (Varjosalo et al., 2013). One common feature shared by the MAPK/CDK family is that their substrates have a proline at +1 position (Pinna and Ruzzene, 1996). Compared to the pH 7.4 control, pH 6 reduced the phosphorylation of MAPK/CDK substrates (pMAPK/CDKSS) by 20%. When normalized to pH 7.4 from the same set of culture, the relative phosphorylation of pMAPK/CDKSS in pH 6 was 80 ± 3.4% (*p* = 0.0022, 2-tailed *t*-test) (Figure 3A). pH 6 treatment had no significant effect on two other members of this family, as shown by the antibodies recognizing phospho-CDK substrate (pCDKSS) and phospho-Casein Kinase 2 substrate (pCK2SS) (Figures 3B,C). We next examined ataxia telangiectasia mutated kinase (ATM) and ataxia telangiectasia and Rad3-related kinase (ATR), which are important for genome stability, DNA repair, and cell cycle control (Cimprich and Cortez, 2008; Burger et al., 2019). Similar to the CMGC family, pH 6 had no significant effect on phospho-ATM/ATR substrate [(pS/pT)QG] (Figure 3D). We also examined the Phospho-Thr-X-Arg (pTXR) motif, which associates with the activation of multiple pathways, including Ras, Erk, and Leucine-rich repeat kinase 2 (Pearson and Kemp, 1991; Choi et al., 2015; Nichols et al., 2019). 1-h pH 6 had no significant effect on pTXR signal (Figure 3E).

### Acidosis-Induced CaMKII Activation Does Not Dependent on GPR68

**Figure 4:**
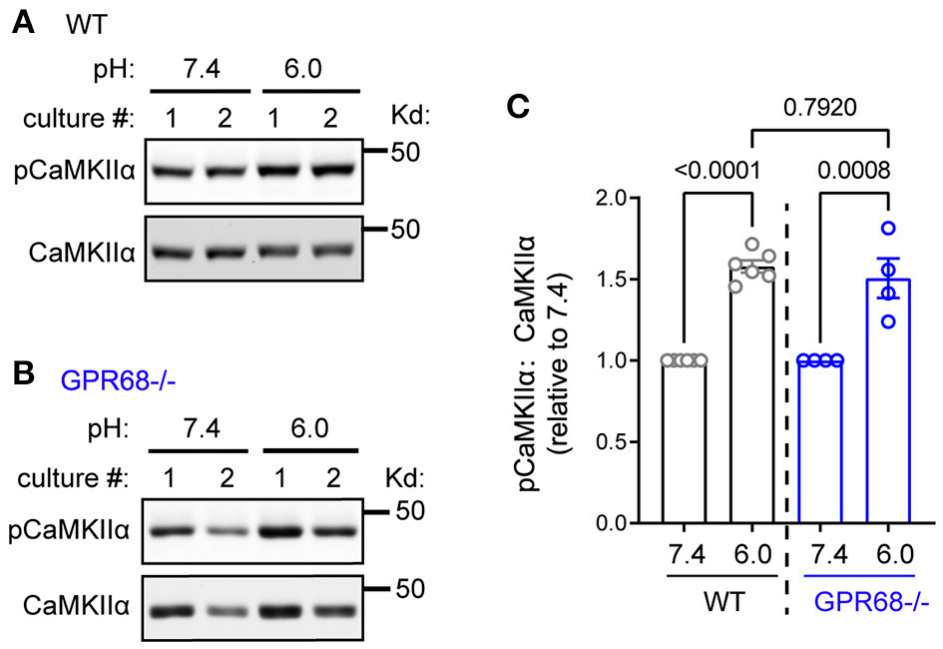
pH 6.0-induced CaMKIIα phosphorylation does not depend on GPR68. Representative Western blot and quantification of extracellular pH 6.0-induced immunoreactivity of pCaMKIIα in WT **(A,C)** and GPR68^−/−^ **(B,C)** hippocampal slices, on acid-induced phosphorylation of Thr286 on CaMKIIα **(A,C)** in WT hippocampal slices. Organotypic hippocampal slices were treated with pH media as indicated, lysed, and analyzed by Western blot. Blots and images were representative from 4 to 6 different cultures/pups (see Methods or Figure 2 Legends for additional clarification). *P*-values were from 2-way ANOVA with Tukey's correction.

In previous studies, acidic stimulation leads to rapid increase in intracellular calcium (Xiong et al., 2004; Yermolaieva et al., 2004; Huang et al., 2008; Wang et al., 2020b). This result suggests that acidosis activates calcium-dependent pathways. Since one dominant calcium target in neurons is CaMKII, we asked whether a prolonged acidosis activates CaMKII. After 1-h pH 6, CaMKIIα exhibited a significant increase (158 ± 3.8%, *p* < 0.0001, student's *t*-test) in phosphorylation of Thr286, the key phosphorylation site for its activation (Figures 4A,C). We recently showed that GPR68, a proton-sensing G protein-coupled receptor (GPCR), was highly expressed in brain neurons (Wang et al., 2020b; Xu et al., 2020). Since GPR68 can mediate calcium signaling and do not readily desensitize (Ludwig et al., 2003; Mohebbi et al., 2012; Wei et al., 2015), we asked whether this increase in CaMKII phosphorylation depends on GPR68. In GPR68^−/−^ slices, pH 6 treatment induced a similar increase in CaMKIIα phosphorylation (151 ±12.2%, *p* = 0.0008) (Figures 4B,C). The relative increase was not statistically different between WT and GPR68^−/−^ slices.

### GPR68 Contributes in Part to Acidosis-Induced Activation of PKA and Akt

**Figure 5:**
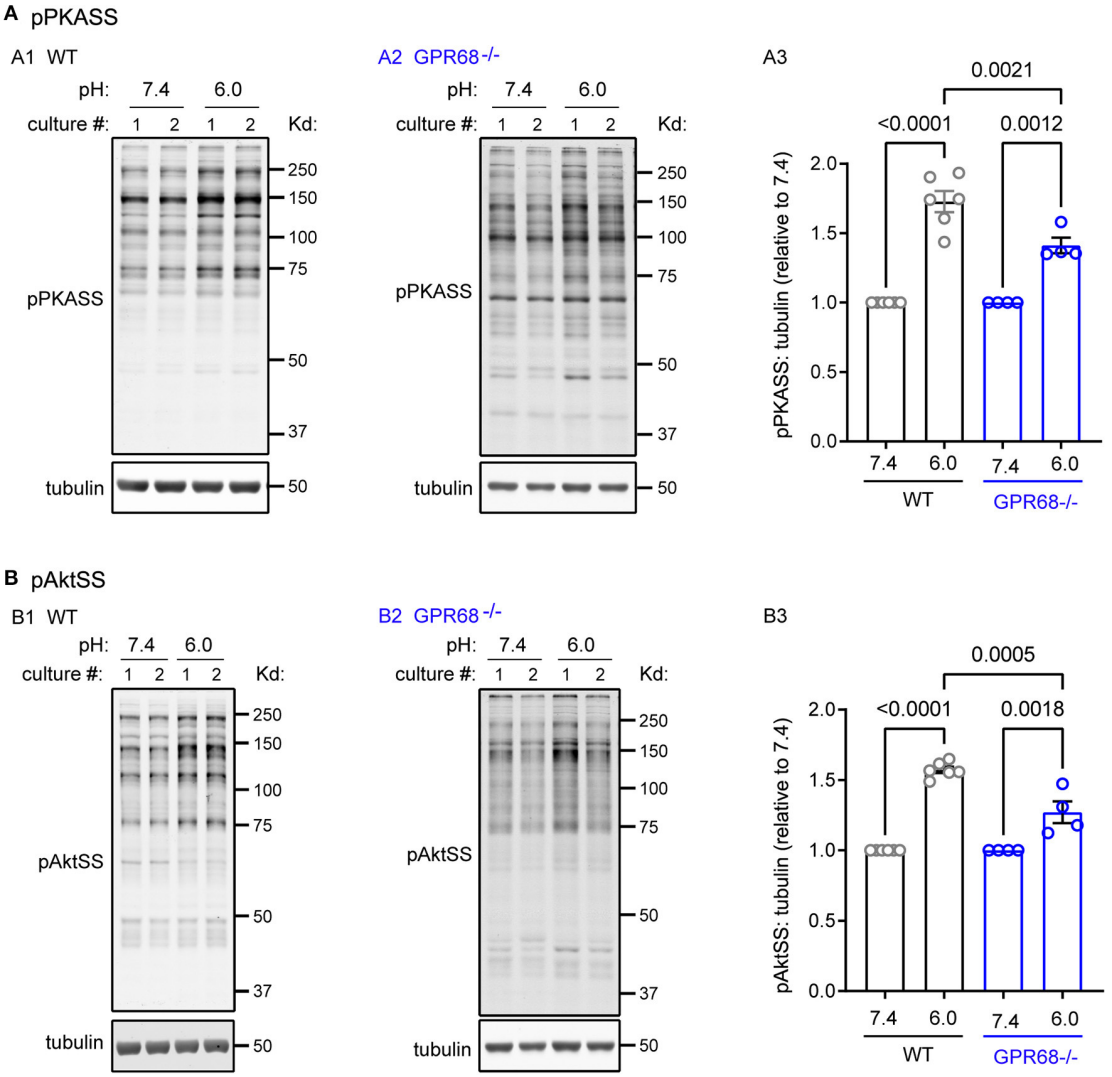
Acidosis increases the phosphorylation of PKA and Akt substrates. Representative Western blot and quantification of 1-h pH 6-induced phosphorylation of **(A)** PKA substrates (pPKASS) and **(B)** Akt substrates (pAktSS) in WT and GPR68^−/−^ hippocampal slices. Blots and images were representative from 4 to 6 different cultures/pups (see Methods or Figure 2 Legends for additional clarification). *P*-values were from 2-way ANOVA with Tukey's correction.

The AGC kinase group, which PKC belongs to, is important for various aspects of cell proliferation and metabolism (Arencibia et al., 2013; Leroux et al., 2018). We focused on two other kinases which are important for cell survival: Akt and PKA. To determine their activation, we used antibodies which recognize the phospho-substrates of Akt (pAktSS) and PKA (pPKASS). One hour pH 6 treatment led to significant increase in immunoreactivity of both substrate antibodies (Figures 5A1,B1). When normalized to pH 7.4, the relative phosphorylation in pH 6 was 173 ± 7.6% for pPKASS (*p* < 0.0001) and 157 ± 2.2% for pAktSS (*p* < 0.0001) (Figures 5A3,B3). To determine whether GPR68 mediates the activation, we examined GPR68^−/−^ slices. In the knockout slices, pH 6 increased the phosphorylation of PKASS and AktSS (Figures 5A2,B2). However, the relative phosphorylation was 141 ± 5.6% for PKASS and 127 ± 7.8% for AktSS; both smaller as compared to those of WT slices (Figures 5A3,B3).

### Acidosis-Induced Tyrosine Phosphorylation Is GPR68-Dependent

**Figure 6:**
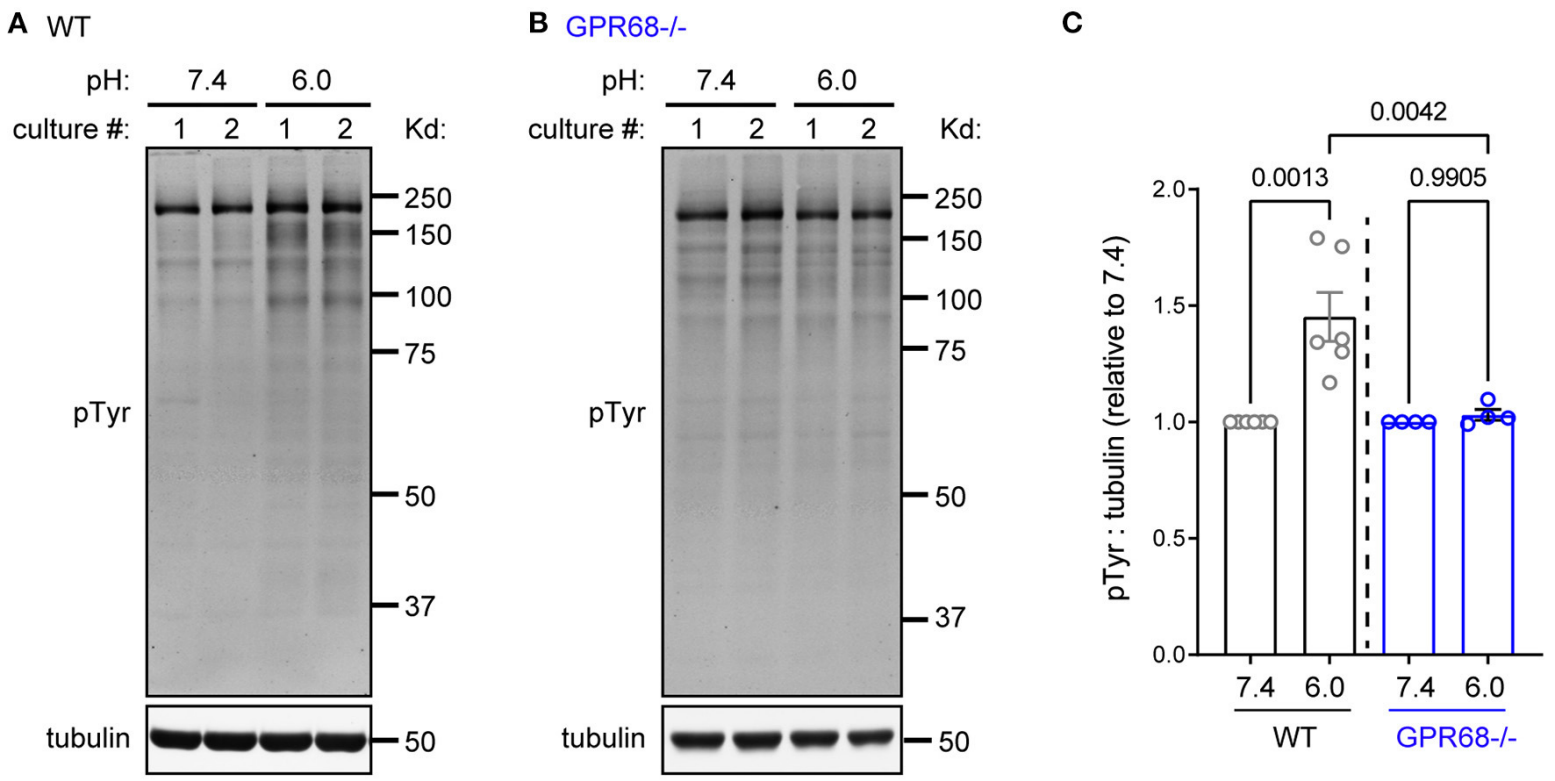
GPR68 mediates acidosis-induced tyrosine phosphorylation. Representative Western blot **(A,B)** and quantification **(C)** of 1-h pH 6-induced tyrosine phosphorylation in WT **(A)** and GPR68^−/−^ **(B)** hippocampal slices. Blots and images were representative from 4 to 6 different cultures/pups (see Methods or Figure 2 Legends for additional clarification). *P*-values were from 2-way ANOVA with Tukey's correction.

All above analysis focused on serine/thereonine kinases. Tyrosine phosphorylation, however, plays diverse roles in a wide range of cellular functions in the central nervous system (Mattson, 1997; Skaper et al., 2001). Therefore, we asked whether acidosis also regulates tyrosine phosphorylation in brain slice, using a phospho-Tyrosine (pY) antibody. pH 6 increased tyrosine phosphorylation by 145 ± 10.5% (Figure 6A). In contrast, pH 6 did not lead to significant changes in pY signals in the GPR68^−/−^ slices (103 ± 2.3%) (Figure 6B).

### Time-Dependent Acid-Induced pPKCSS and pCaMKIIα Increase in Acute Hippocampal Slices

**Figure 7:**
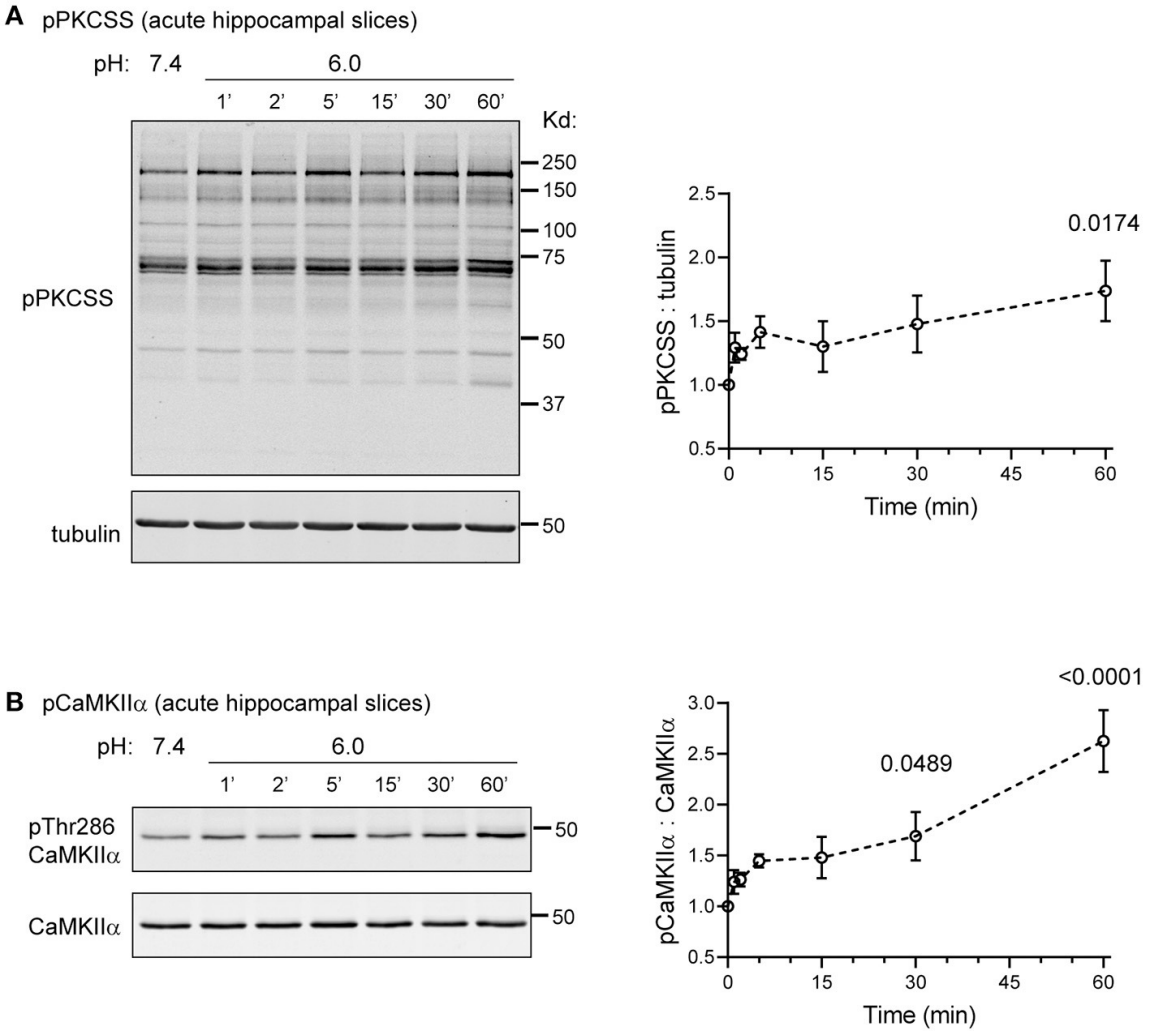
Acidosis-induced pPKCSS and pCaMKIIα changes in acute hippocampal slices. Representative Western blot and quantification of pH 6-induced pPKCSS **(A)** and pCaMKIIα **(B)** signals in hippocampal slices acutely isolated from young adult mice. Acute slices were prepared from 7 to 9 week old WT male mice as described in Methods. Following recovery in ACSF, the slices were treated with pH 6 for the indicated time, lysed, and blotted for the antibodies indicated. Each animal generated enough slices for the entire time course. Summary plot was from 4 animals. Each data point was compared with pH 7.4 (the “0” time point on graph) (1-way Anova with Dunnett's *post-hoc* correction). For time points exhibited significant differences, the actual *p*-value was reported on the graph.

The above analysis examined phosphorylation changes in organotypic hippocampal slices. To gain more insights regarding acid signaling in more mature brain, we further examined acutely prepared hippocampal slices from 7 to 9 week old WT mice. We used a similar protocol as that typically used for physiological recordings (Xu et al., 2020). Following sectioning and recovery, we either incubated the slices in pH 7.4 control buffer, or for different time points in pH 6.0. We then lysed the slices and performed Western blot analysis. In this experiment, we focused on pPKCSS and pCaMKIIα because previous studies have demonstrated their importance in acidosis-induced injuries (Katsura et al., 1999; Mattiazzi et al., 2007; Wang et al., 2020b). pH 6 induced time-dependent increase in pPKCSS and pCaMKIIα signals (Figure 7). Due to relatively small N (=4), the early time points were not statistically significant following *post-hoc* correction. Nevertheless, the trend of increase was apparent starting at 2 min after pH 6 treatment. At 60 min, pH 6-induced increase in phosphorylation was significant for both pPKCSS and pCaMKIIα.

## Discussion

Protein kinases play key roles in signal transduction in eukaryotic cells (Manning et al., 2002; Wilson et al., 2018). In previous studies, acidosis can activate a wide range of kinases, including PKA, PKC, Akt, Rho kinase, MAPK, and CaMKII (Mattiazzi et al., 2007; Pereverzev et al., 2008; Riemann et al., 2011; Huang W. Y. et al., 2015; Krewson et al., 2020; Ogasawara et al., 2020; Wang et al., 2020b). However, most of these studies examined non-neuronal cells. Here, we studied organotypic hippocampal slices, which maintain *in vivo* circuitry and provide a good *ex vivo* system to study brain physiology (Hutter-Schmid et al., 2015; Croft et al., 2019). Our data here showed that acidosis induced the activation of AGC family kinases and CaMKII, attenuated the phosphorylation of MAPK/CDK substrates, but had little effect on ATM/ATR, CDK, or CK2 kinases. Further, our data from GPR68^−/−^ demonstrated that GPR68 contributes, at least in part, to acid-induced activation of AGC kinases.

The activation of CaMKII and PKC by acidosis is consistent with previous reports (Kurihara et al., 2004; Zha et al., 2006; Sherwood et al., 2009; Wang et al., 2020b). In previous studies, the activation of ASIC1a leads to calcium increase and CaMKII activation (Xiong et al., 2004; Yermolaieva et al., 2004; Zha et al., 2006; Du et al., 2014a). GPR68 activation, in contrast, primarily couples to Gq, and induce calcium increase and PKC activation (Ludwig et al., 2003; Wei et al., 2015; Glitsch, 2019; Wang et al., 2020b). However, GPR68 deletion had little effect on acidosis-induced CaMKII phosphorylation, suggesting that the relative contribution of GPR68 to the calcium-CaMKII pathway, at least at this persistent stage, is limited. Given that ASICs exhibit desensitization in acidotic condition, we speculate that additional receptor or intracellular targets may contribute to chronic acidosis-induced CaMKII activation.

Similar to our previous observation at pH 6.5 (Wang et al., 2020b), GPR68 deletion abolished pH 6-induced phosphorylation of PKC substrates. This result indicates that GPR68 is one main mediator of acidosis-induced PKC activation. Further, our survival study here, consistent with our previous data in cortical slices, demonstrates a critical role of PKC in mediating a protective function under acidotic condition. While we do not know the specific PKC subtypes involved here, previous studies have shown that PKC epsilon is important for neuroprotection in brain injury (Perez-Pinzon et al., 2005).

Besides CaMKII and PKC, our data imply that 1 h of acidosis (pH 6) may activate PKA and Akt, which are two AGC kinases important for neuronal survival. The exact mechanism for acidosis-induced PKA and Akt activation in hippocampal slices remains unclear. Previous studies in heterologous cells further showed that GPR68 can couple to alternative pathways such as PKA (Pereverzev et al., 2008; Wiley et al., 2018). However, GPR68 deletion only has partial effect on either pPKASS or pAktSS signals, suggesting that additional effectors are involved. GPR65, another proton-sensitive GPCR, has only limited expression in the brain, both in overall expression level and the number of GPR65 expressing cells (Vollmer et al., 2016; Sato et al., 2020; Zhou et al., 2021). GPR65 thus is unlikely to alter phosphorylation at a global (i.e., whole slice) level. GPR4 is present throughout endothelial cells and in restricted number of neurons (Hosford et al., 2018; Wenzel et al., 2020). This expression pattern suggests that GPR4-dependent signaling may contribute to the global phosphorylation changes. In previous studies, mostly in peripheral or heterologous cells, GPR4 exhibit relatively promiscuous signaling through Gs, Gq, and G_12/13_ (Chen et al., 2011; Wyder et al., 2011; Hosford et al., 2018; Wang et al., 2018; Krewson et al., 2020; Wenzel et al., 2020). Thus, it is possible that GPR4-dependent signaling mediates part of acidosis-induced PKA and Akt activation observed here. It will be of future interest to study GPR4^−/−^ slices to assess its contribution. Another important consideration is that extracellular acidosis leads to intracellular acidosis, and vice versa (Siesjo, 1982; Boedtkjer, 2018). With a prolonged acidosis, especially after hours of incubation, acidosis will alter signaling through both directly activating proton receptors and modulating intracellular effectors. For example, mitochondria are one critical target of acidosis and play important roles in metabolic and apoptotic signaling (Siesjo et al., 1996; Pagliaro and Penna, 2011). These data suggest that the changes in signaling, especially following a prolonged phase of pH reduction, likely will involve both extracellular and intracellular mediators.

How acidosis attenuated the phosphorylation of MAPK/CDK substrates remains unclear. Since there were no apparent changes in pCDKSS signal (Figure 3B), we speculate that this result suggests a reduction specifically in the activities of MAPKs (Erk/JNK/p38) as opposed to that of CDKs. The most striking reduction in Figure 3A was the bands around 60–65 kD. We currently do not know the identity of this/these proteins. However, several proteins that MAPKs can regulate and have important functional impact in neuronal injury are within this molecular weight range: ATF-2, Tau, p70 S6K, and NFkB (Zhang et al., 2001; Bjorkblom et al., 2008; Bi et al., 2017; Saha et al., 2020).

Since previous studies have shown GPR68 signals through multiple serine/threonine pathways (Pereverzev et al., 2008; Tomura et al., 2008; Wiley et al., 2018; Maeyashiki et al., 2020), it is provocative to observe that acidosis induced phosphorylation of tyrosine in a GPR68-dependent manner. Most of the increases occurred between molecular weight 75–250 kD (Figure 6). It is of interest to note that multiple receptor tyrosine kinases, including TrkB, exhibit apparent molecular weight at ~90–200 kD. Insulin receptor substrate 1 and 2 (IRS 1 and 2), which mediates the signaling of multiple receptor tyrosine kinase receptors, run at about 180 kD (Yamada et al., 1997; Schubert et al., 2003). While the exact mechanism warrants further study, this result suggests that the activation of tyrosine kinases play an important role in GPR68-mediated function. Given the importance of tyrosine phosphorylation in neuron function and neuronal survival (Skaper et al., 2001), it will be important to further explore the downstream effectors and functional consequences of GPR68-induced tyrosine phosphorylation in a future study.

In summary, our data revealed that acidosis preferentially activates AGC kinases, CaMKII, and tyrosine phosphorylation, had little effect on CDK, CK2, and ATM kinases in hippocampal slices. GPR68 mediates acidosis-induced PKC activities and tyrosine phosphorylation, and contributes in part to PKA and Akt activation. This finding raises the potential of crosstalk among the pathways. In this study, we focused our analysis in organotypic hippocampal slices at a single time point (1-h) and one pH value, which facilitates the profile-type analysis of phosphorylation. In acute slices, we found time-dependent increase in pPKCSS and pCaMKII signals. These results together suggest a good qualitative consensus between organotypic and acute (adult) hippocampal slices. To gain better understanding of the dynamics of neuronal acid signaling, it will be necessary to perform additional systematic studies and examine additional pH values and multiple time points in the same (i.e., organotypic or acute) system. Moreover, further analysis is necessary to determine which GPR68-independent pathways contribute to acid signaling at the chronic stage. Nevertheless, our data indicate that a network of kinases work in concert to determine the outcome of various neurological diseases which lead to persistent pH reduction.

## Data Availability Statement

The original contributions presented in the study are included in the article, further inquiries can be directed to the corresponding author.

## Ethics Statement

The animal study was reviewed and approved by Animal Care and Use Committee at University of South Alabama.

## Author Contributions

GZ performed the experiment, analyzed the data, and wrote the manuscript. X-mZ designed the study, performed pilot experiment, and wrote the manuscript. Both authors reviewed the manuscript.

## Conflict of Interest

The authors declare that the research was conducted in the absence of any commercial or financial relationships that could be construed as a potential conflict of interest.
